# Asthma rehabilitation at high vs. low altitude: randomized parallel-group trial

**DOI:** 10.1186/s12890-019-0890-y

**Published:** 2019-07-24

**Authors:** Stéphanie Saxer, Simon R. Schneider, Paula Appenzeller, Patrick R. Bader, Mona Lichtblau, Michael Furian, Ulan Sheraliev, Bermet Estebesova, Berik Emilov, Talant Sooronbaev, Konrad E. Bloch, Silvia Ulrich

**Affiliations:** 1 Department of Pulmonology, UniversityHospital Zurich, Rämistrasse 100, CH-8091 Zurich, Switzerland; 2 Department of Health Sciences & Health Policy, University of Lucerne, Lucerne, Switzerland; 3 National Center for Cardiology and Internal Medicine, Bishkek, Kyrgyzstan

**Keywords:** Asthma, Pulmonary rehabilitation, Altitude

## Abstract

**Background:**

To investigate the effect of asthma rehabilitation at high altitude (3100 m, HA) compared to low altitude (760 m, LA).

**Methods:**

For this randomized parallel-group trial insufficiently controlled asthmatics (Asthma Control Questionnaire (ACQ) > 0.75) were randomly assigned to 3-week in-hospital rehabilitation comprising education, physical-&breathing-exercises at LA or HA. Co-primary outcomes assessed at 760 m were between group changes in peak expiratory flow (PEF)-variability, and ACQ) from baseline to end-rehabilitation and 3 months thereafter.

**Results:**

50 asthmatics were randomized [median (quartiles) LA: ACQ 2.7(1.7;3.2), PEF-variability 19%(14;33); HA: ACQ 2.0(1.6;3.0), PEF-variability 17%(12;32)].

The LA-group improved PEF-variability by median(95%CI) -7%(− 14 to 0, *p* = 0.033), ACQ − 1.4(− 2.2 to − 0.9, *p* < 0.001), and after 3 months by − 3%(− 18 to 2, *p* = 0.103) and − 0.9(− 1.3 to − 0.3, *p* = 0.002). The HA-group improved PEF-variability by − 10%(− 21 to − 3, *p* = 0.004), ACQ − 1.1(− 1.3 to − 0.7, *p* < 0.001), and after 3 months by − 9%(− 10 to − 3, *p* = 0.003) and − 0.2(− 0.9 to 0.4, *p* = 0.177). The additive effect of HA vs. LA directly after the rehabilitation on PEF-variability was − 6%(− 14 to 2), on ACQ 0.3(− 0.4 to 1.1) and after 3 months − 5%(− 14 to 5) respectively 0.4(− 0.4 to 1.1), all *p* = NS.

**Conclusion:**

Asthma rehabilitation is highly effective in improving asthma control in terms of PEF-variability and symptoms, both at LA and HA similarly.

**Trial registration:**

Clinicaltrials.gov: NCT02741583, Registered April 18, 2016.

**Electronic supplementary material:**

The online version of this article (10.1186/s12890-019-0890-y) contains supplementary material, which is available to authorized users.

## Background

Asthma is a major global health problem affecting over 300 million people worldwide with increasing prevalence in developing countries [1]. Asthma causes respiratory symptoms (mainly cough, dyspnea), limitation of activity and exacerbations that may require emergency treatment [1].

The current Global Initiative for Asthma (GINA) has outlined that patients with asthma should be managed, according to their level of asthma control, which includes symptoms during the day, symptoms during the night, use of reliever medication, limitation of daily activity, pulmonary function and exacerbations [1].

Many patients with asthma can be adequately controlled with bronchodilators in combination with inhaled corticosteroids. However, some asthma patient suffer from refractory disease despite medical therapy [2] and even controlled patients have to be prepared for flare-ups. Besides the pharmacological treatment, rehabilitation programs might be beneficial for asthma patients. The GINA guidelines state to advice all patients with chronic airflow disease for rehabilitation, whereas high altitude treatment has evidence level C [1].

Rehabilitation contains not only physical training and breathing exercises but also education, including smoking cessation [3, 4] aiming to deliver a profound insight into the disease and the ability to adequately react on exacerbations [5].

For decades, asthma patients have been sent to altitude clinics aiming to improve asthma control [6, 7]. At high altitude there is less allergen exposure [8, 9], e.g. house dust mites do not exist > 1600 m above sea level (asl). A factor that might also mitigate a potential beneficial effect of altitude exposure on asthma control is the dry and generally less polluted air resulting in less inflammation [10, 11] and improved respiratory function [12]. In addition, the decreased air density at altitude leads to reduced airway resistance [13], which might additionally help to increase exercise capacity for asthmatics at altitude. The mountain environment may also signify less psychosocial stress for many patients having the opportunity to leave their everyday environment behind and enjoy a pleasant stay at altitude. Stress is associated with reduced sensitivity to endogenous glucocorticosteroids and chronic psychological stress has been associated with asthma exacerbation, whereas stress reduction seems to improve asthma control [14, 15].

Kyrgyzstan is a lower middle- income country with less access to advanced drug therapies [16]. Thus, allergen avoidance along with an intense asthma education and rehabilitation program is potentially of even greater importance in order to avoid disease exacerbations or chronification due to low asthma control.

As in the Swiss Alps, since many years asthma patients have been taken to Tuja Ashu, a high-altitude clinic located at 3100 m asl in the mountain area of the Kyrgyz Republic to improve asthma control. However, randomized controlled trials, which compare the effect of asthma rehabilitation at high compared to low altitude are completely lacking. Whether the reported positive effect of asthma rehabilitation at Alpine resorts is due to hypobaric hypoxic environment at altitude with less allergen exposure or due to the comprehensive rehabilitation program, including patient education and exercise or both is not clear to date.

We aimed to study a specific in-patient asthma rehabilitation program and its effectiveness on asthma control and investigated the effects of performing the rehabilitation at the Tuja Ashu high altitude clinic (HA), 3100 m, in comparison to the same program performed at low altitude in Bishkek (LA), 760 m. We hypothesized that a 3-week rehabilitation at high altitude improves PEF-variability and ACQ compared to a low altitude 3-week rehabilitation. Additionally, we hypothesized the effects would be sustained at 3 months follow-up.

## Material and methods

### Design

In this randomized parallel-group trial asthmatics were assigned to in-patient rehabilitation either at LA (Bishkek, Kyrgyzstan, 760 m) or HA (Tuja Ashu, Kyrgyzstan, 3100 m), between May and October 2016. The study was approved by the local Kyrgyz Ethics Committee (01–8/151) and the Ethics Committee Zurich (2016–00076).

### Participants

Adults (≥18 years) diagnosed with atopic (positive prick test) or non-atopic asthma according to the GINA-guidelines were eligible if there disease was not well controlled, defined as asthma control questionnaire (ACQ) score > 0.75 [17] and that have given written informed consent. Patients living permanently at > 1′000 m asl, heavy smokers (≥20 cigarettes/day), patients with a history of acute mountain sickness (AMS) at altitudes < 3′100 m, or with serious concomitant diseases (Additional file 1) were excluded. Study physicians recruited patients in the National Center for Cardiology and Internal Medicine in Bishkek (Kyrgyzstan).

### Intervention

The 3-week rehabilitation program (for detailed description see Additional file 1) was identical for both groups and consisted of patient education, endurance training, muscle strength training, breathing exercises and guided walks (each 5x/week for 30-45 min and a total duration of 5 h/day). All patients received an asthma action plan containing instructions on recognition of worsening asthma control and on suggested actions [1]. Patients from the HA group were transferred via minibus, to the high altitude clinic and back to Bishkek, respectively.

### Outcomes

All outcomes were assessed at 760 m asl.

The co-primary outcomes were the differences of the changes between the two groups (LA vs. HA) in the score of the ACQ and the PEF-variability from baseline to the end of the 3-week rehabilitation and to 3-month follow-up (FU). The PEF-variability was computed as [(day’s highest-day’s lowest)/mean of day’s highest+lowest] [1]. PEF measures were made four times daily.

Secondary outcomes were the percentage of patients with well controlled or partially controlled asthma (ACQ score < 1.5), the between-group differences of the change in the following assessments to end of rehabilitation and FU: forced expiratory volume in 1 s (FEV1), PEF-variability, asthma-related quality of life (AQLQ), generic quality of life assessed by the short-form 36 questionnaire (SF-36), exercise performance assessed by the sit-to-stand (STS), 6 min walking distance (6MWD), airway inflammation assessed by the exhaled nitric oxide (FeNO) [18] and blood eosinophils and hemoglobin concentration.

The HA-group additionally filled out the Environmental Symptom Questionnaire cerebral score (AMS-c) at the second day at altitude to assess safety of altitude exposure symptoms of acute mountain sickness [19].

### Sample size estimation

With an estimated standard deviation of the ACQ-score of 1 [7] and a minimal clinical important difference of 0.5 [20], 16 patients needed to be included per group to achieve a power of 0.8 with an alpha of 0.05. For the same power of the co-primary endpoint PEF-variability, we assumed a difference of 3% in PEF-variability with a standard deviation of 3% between the groups again indicating 16 patients. Thus, to account for drop-outs, we aimed to include at least 20 patients per group.

A list of the eligible patients was sent to an independent coordinator who randomly allocated patients to rehabilitation at LA or HA by a computer program (Stata 14.0, StataCorp, College Station, Texas, USA) with the plugin “rct_minim”) providing minimisation with regard to atopy [21]. Blinding of the intervention (rehabilitation and altitude) was intrinsically not possible.

### Data analysis

The analysis was performed on data from all participants undergoing rehabilitation at one of the two locations. Data are summarized as median (interquartile range, IQR). The primary and secondary outcomes were evaluated with the Wilcoxon test for paired samples within group and the Mann-Whitney-U-Test for the difference between groups. Difference between the change in PEF-variability and change in ACQ from baseline to 3 weeks and from baseline to 3 months follow-up were calculated and presented as median differences with 95% confidence intervals. A *p* < 0.05 was considered statistically significant.

Linear regression analysis was used to estimate the effect of the patients’ characteristics on the change of the PEF-variability. Statistical analyses were conducted with SPSS 22 (SPSS, Chicago, Illinois, USA).

### Data reporting

The data is reported in adherence with the CONSORT guidelines.

## Results

**Fig. 1:**
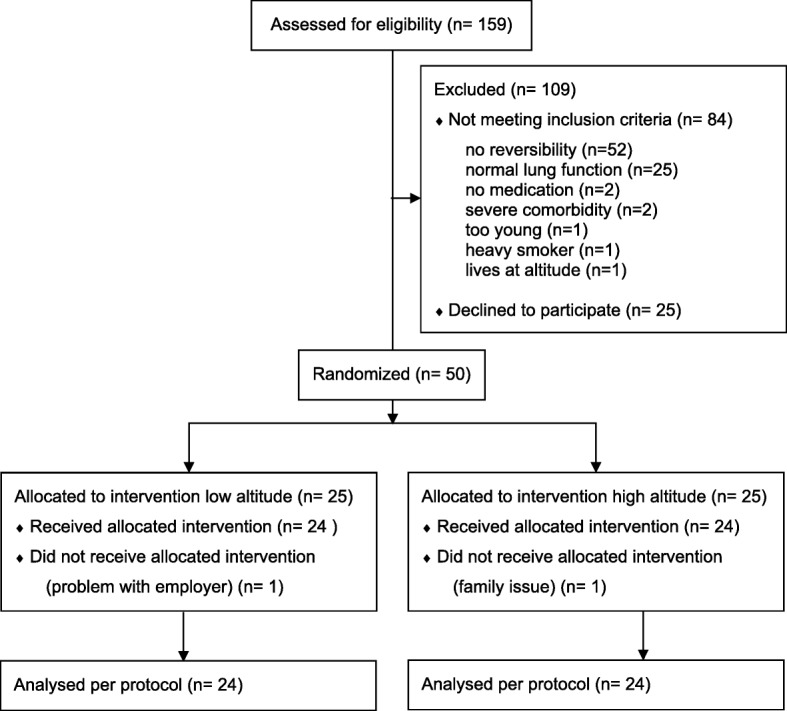
Study flow chart

Of 159 eligible patients 50 were included in the study: 25 per group with one patient in each group not receiving the intervention (one for professional and one family reasons), see study flow chart Fig. 1. Therefore, the analysis was performed per protocol on data from all participants undergoing rehabilitation. Baseline characteristics are shown in Table 1.

**Table 1:** Baseline characteristics

	Low altitude (760 m) rehabilitation group	High altitude (3100 m) rehabilitation group
Number of participants (Females)	25 (18, 72%)	25 (16, 64%)
Age, years	47 (34;53)	43 (33;49)
Body mass index, kg/m^2^	24 (23;27)	26 (23;29)
Non-smokers, *n* (%)	25 (100)	25 (100)
Atopy, *n* (%)	23 (92)	23 (92)
Peak expiratory flow, L/min	311 (274;378)	326 (261;368)
FeNO, ppb	37 (26;46)	51 (37;73)

*Note:* Data are given as median (quartiles) or numbers (%)

*Abbreviations: FeNO* exhaled nitric oxid

### Primary outcomes

**Fig. 2:**
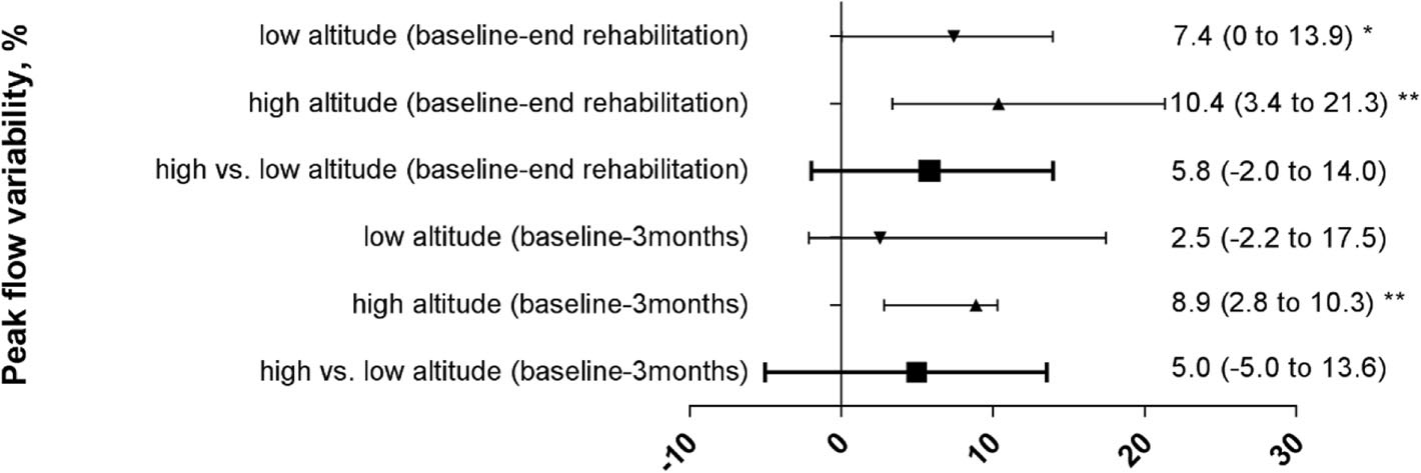
Peak flow variability of both groups, median changes (95%CI) from baseline to end rehabilitation after 3 weeks and 3 months follow up and median differences (95%CI) between groups

**Fig. 3:**
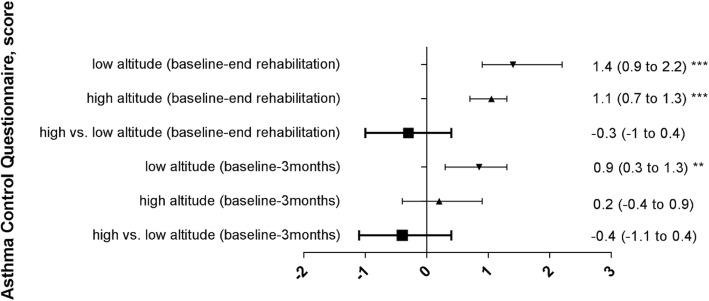
Asthma control of both groups, median changes (95%CI) from baseline to end rehabilitation after 3 weeks and 3 months follow up and median differences (95%CI) between groups

At baseline, the elevated values of ACQ and PEF-variability confirmed that the asthma was poorly controlled according to selection criteria. Both outcomes significantly improved during the 3-week rehabilitation program at LA although PEF-variability was reduced to below 10% in the HA-group only. After 3 months, the improvements in ACQ were maintained in both groups while PEF-variability remained significantly below baseline and below 10% in the HA-group only. Moreover, the absolute values of PEF-variability at 3 weeks and 3 months were lower in the HA-group compared to the LA-group. There was no significant difference between groups (HA vs. LA) with regard to the changes of the ACQ and the PEF-variability from baseline to end of rehabilitation at 3 weeks and FU at 3 months (Table 2 and Figs. 2, 3).

**Table 2:** Primary endpoints assessed at low altitude (760 m)

	Low altitude rehabilitation group			High altitude rehabilitation group			Between group difference of changes from BL to 3 weeks	Between group difference of changes from BL to 3 months
	Baseline	End of rehabilitation (3 weeks)	Follow-up (3 months)	Baseline	End of rehabilitation (3 weeks)	Follow-up (3 months)		
PEF-Var.	19 (14;33)	15 (8;23)	15 (9;24)	17 (12;32)	6 (4;9)#	8 (5;14)#		
change		−7.4 (−13.9 to 0)*	−2.5 (-17.5 to 2.1)		−10.4 (−21.3 to −3.4)**	−8.9 (-10.3 to -2.8)**	− 5.8 (− 14 to 2)	− 5 (− 13.6 to 5.0)
ACQ	2.7 (1.7;3.2)	0.8 (0.4;1.6)	1.4 (0.9;2.1)	2.0 (1.6;3.0)	0.9 (0.4;1.6)	1.6 (0.9;3.0)		
change		-1.4 (-2.2 to −0.9)***	− 0.9 (− 1.3 to −0.3)**		−1.1 (−1.3 to − 0.7)***	−0.2 (-0.9 to 0.4)	0.3 (-0.4 to 1.1)	0.4 (− 0.4 to 1.1)

*Note:* Data are given as median (quartiles) and median difference (confidence interval)

*Abbreviations: PEF-Var.* Peak-flow variability, %, *ACQ* Asthma Control questionnaire. *: *p* < 0.05; **: *p* < 0.01; ***: *p* < 0.001 tested with Wilcoxon test for paired samples (baseline vs. 3 weeks follow up and baseline vs. 3 months follow up). #: *p* < 0.05 vs. low altitude group

### Secondary outcomes

The percentage of patients with well controlled or partly controlled asthma (ACQ < 1.5) [17] increased in the LA-group from 20, to 83% (*p* < 0.001) after the rehabilitation and to 54% (*p* = 0.003) at 3 months; corresponding values in the HA-group were 16, 71% (*p* < 0.001) and 48% (*p* = 0.008) respectively).

At the end of the 3-week rehabilitation almost all secondary outcomes, including the 6MWD, STS, lung function and quality of life assessed at 760 m significantly improved in both groups (Tables 3 and 4). Asthma control measured by the use of reliever medication (ACQ question number 6) significantly improved with rehabilitation (LA *p* = 0.025, HA *p* = 0.002) and to a similar extent in both groups (Additional file 1: Table S1).

**Table 3:** Secondary endpoints

	Low altitude			High altitude			Between group difference of changes from BL to 3 weeks	Between group difference of changes from BL to 3 months
	Baseline	End of rehabilitation (3 weeks)	Follow-up (3 months)	Baseline	End of rehabilitation (3 weeks)	Follow-up (3 months)		
FEV1, %pred.	61 (42;77)	96 (54;80)*	71 (58;83)*	64 (59;71)	78 (67;83)***	72 (58;85)*	3.6 (−4.9 to 10.9)	1.7 (-7.3 to 9.6)
FVC, %pred.	83 (63;93)	87 (79;96)*	87 (77;94)	82 (74;92)	91 (83;98)**	84 (78;95)	1.5 (−5.5 to 10.2)	−.45 (-7.0 to 6.1)
FEV1/ FVC, pre	.63 (.52;.69)	.63 (.53;.70)	.67 (.54;.70)*	.67 (.59;.71)	.70 (.63;.75)***	.69 (.6;.75)**	.01 (−.03 to .05)	.01 (−.04 to .06)
6MWD, m	490 (452;510)	521 (493;549)***	504 (486;536)*	536 (491;571)	571 (522;624)**	570 (517;607)	−2 (-24 to 22)	4 (-26 to 31)
STS, repetitions	21 (19;24)	24 (21;26)**	25 (22;28)**	25 (23;28)	36 (30;40)***	30 (27;39)***	**7 (4 to 11)**	**4 (0 to 7)**
FeNO, ppb	37 (25;58)	19 (36;51)	–	51 (35;76)	27 (23;52)**	–	**−18 (−36 to 0)**	
Hemoglobin, g/l	139 (131;150)	139 (125;145)		142 (127;158)	145 (138;166)		**7 (4 to 11)**	
EOS, 10^9/l	.39 (.28;.63)	.31 (.18;.57)		.38 (.28;.57)	.26 (.18;.45)		−.05 (−.16 to .04)	
EOS, %	6.3 (5.0;9.3)	5.4 (2.7;7.1)*	–	6.2 (5.1;8.6)	3.9 (2.4;6.5)***	–	−1.1 (−2.6 to 0.5)	

*Note:* Data are given as median (quartiles) and median difference (confidence interval)

*Abbreviations: FEV1* forced expiratory volume in 1 s, *FVC* forced vital capacity, *6MWD* 6 min walking distance, *STS* Sit-to-stand, *FeNO* exhaled nitric oxid, *EOS* blood eosinophils

*: *p* < 0.05; **: *p* < 0.01; ***: *p* < 0.001 tested with Wilcoxon test for paired samples (baseline vs. 3 week follow up and baseline vs. 3 months follow up); bold: significant between group differences

**Table 4:** Effect of HA and LA rehabilitation on Quality of Life

	Low altitude			High altitude				
	Baseline	End of rehabilitation (3 weeks)	Follow-up (3 months)	Baseline	End of rehabilitation (3 weeks)	Follow-up (3 months)	Between group difference of change BL-3w	Between group difference of change BL-3 m
AQLQ Score	3.6 (3.1;4.8)	5.5 (4.4;6.4)***	5.1 (3.8;5.9)***	3.9 (3.1;4.6)	5.6 (4.4;6.3)***	4.9 (3.5;6.2)*	−.0 (−.7 to .8)	−.5 (−1.6 to .3)
AQLQ Activity limitation	4.3 (3.5;5.1)	5.7 (4.4;6.3)**	5.0 (4.3;5.9)***	4.4 (3.5;5.0)	5.9 (4.8;6.4)***	5.0 (3.8;6.4)**	.0 (−.7 to .7)	−.1 (−.7 to .6)
AQLQ Symptoms	3.8 (3.3;4.9)	6.0 (4.9;6.5)***	5.5 (4.0;6.0)**	3.7 (3.1;4.8)	5.4 (4.4;6.6)***	5.5 (3.2;6.5)*	−.2 (−1 to .7)	−.5 (−1.9 to .4)
AQLQ Emotional function	3.4 (2.5;4.5)	5.0 (4.1;6.0)**	4.7 (3.7;6.4)***	3.6 (2.7;4.6)	5.3 (3.8;6.6)***	4.8 (2.8;6.0)**	.0 (−.8 to .8)	−.4 (−1.4 to .4)
AQLQ Environ-mental stimuli	3.3 (2.1;4.0)	5.0 (3.2;6.2)**	4.3 (3.3;5.5)**	3.0 (2.6;4.5)	5.0 (4.6;5.9)***	4.6 (3.0;5.7)**	.0 (−1 to .8)	.0 (−1 to .8)
SF − 36, PCS	40 (35;43)	40 (36;46)	42 (36;47)	50 (43;54)	46 (38;52)	51 (45;54)	-3 (−10 to 3)	−1 (−5 to 4)
SF − 36, MCS	44 (36;49)	41 (34;48)	44 (36;50)	51 (47;56)	48 (41;55)	58 (56;61)	-3 (−12 to 5)	5 (−2 to 11)

*Note:* Data are given as median (quartiles) and median difference (confidence interval)

*Abbreviations: AQLQ* Asthma Quality of Life Questionnaire, *SF-36* Short-form 36, *PCS* Physical component score, *MCS* mental summary score

*: *p* < 0.05; **: *p* < 0.01; ***: *p* < 0.001 tested with Wilcoxon test for paired samples (baseline vs. 3 week follow up and baseline vs. 3 months follow up)

We found that the sit-to-stand test and the FeNO improved significantly more in the HA-group compared to the LA-group. Median changes of other secondary outcomes assessed before and after the 3-week rehabilitation at 760 m were similar at LA or HA (Table 3). Improvements of secondary outcomes were mostly maintained at the follow-up assessment at 3 months. The changed of the STS remained higher in the HA compared to LA.

Furthermore, the HA-group significantly increased the hemoglobin concentration compared to the LA-group from baseline to week 3 [median difference 7 (4 to 11) g/L, *p* > 0.001].

Linear regression analysis (Table 5) revealed that the PEF-variability before the rehabilitation program and the rehabilitation at HA vs. LA were independent predictors of the improvement in PEF-variability [ΔPEF-variability (baseline-end rehabilitation) = − 0.375 + 0.914(baseline PEF-variability) + 0.091(Group); R^2^ 0.666, *p* < 0.001].

**Table 5:** Regression analysis

Dependent variable = Δ Peak flow variability pre-post rehabilitation	Multivariable analysis		
	coefficient	95%CI	*p*
Gender (male =1, female = 2)	.025	−.030 to .079	.371
Age, years	.002	−.001 to .004	.160
Group, (LA = 1 / HA = 2)	.091	.042 to .140	.001
Peak flow variability, % baseline	.914	.718 to 1.111	.000
Peak flow, ml baseline	.011	−.006 to .029	.195
Intercept	−.375	−.607 to −.143	.002

*Note*: *n* = 48. R^2^ was 0.666, *p* < 0.001. CI = confidence interval

*Abbreviations*: *LA* low altitude, *HA* high altitude

### Adverse events and safety measures

Of the 25 patients undergoing rehabilitation at HA, symptoms of AMS defined as score ≥ 0.7 were present in 11 patients in Bishkek (760 m) and 6 patients at altitude with only two having a higher score at HA. One patient needed medical treatment for an AMS with severe headache and a very high AMS score of 4.46.

During the rehabilitation program the following events occurred, which were not directly linked to the training: In the LA rehabilitation group, one patient had a slight cold, one had temporary fever and one had a sore throat; in the HA one patient was treated with acetazolamide for 1 day for relevant AMS (including headache), one had slight headache, one had intermittent diarrhea and one had an asthma exacerbation defined as a decline in PEF of > 12%. None of the patient had to interrupt the whole rehabilitation program for > 1 day due to symptoms.

## Discussion

This is the first randomized controlled trial which investigated the additional effect of undergoing a 3-week rehabilitation program at HA (3100 m) vs. LA (760 m) on asthma control in patients with uncontrolled asthma. We found that a comprehensive 3-week rehabilitation program including education, endurance training, muscle- and breathing exercise significantly improved asthma control both at low and high altitude. The effect of the rehabilitation at HA was not superior compared to the same rehabilitation program at LA in ameliorating asthma control as assessed by the ACQ and PEF-variability. Nevertheless, rehabilitation at HA was well tolerated and led to a better improvement of exercise capacity (STS) and airway inflammation assessed by FeNO. In addition, regression analysis revealed that the overall improvement in PEF-variability from baseline to 3 weeks was better in the HA vs. LA-group when controlling for relevant confounders such as age, gender and the baseline absolute PEF. Thus suggesting that patients with a higher baseline PEF-variability may benefit more from a rehabilitation at altitude.

For many years, asthma patients are taken to HA in order to improve their asthma control in a presumptively cleaner environment with less allergen exposure. The effect of training at altitude (1600 m asl) on asthma control was investigated in a prospective, uncontrolled study by Rijssenbeek-Nouwens and coworkers [7], which showed that sensitized and unsensitized patients improved their ACQ score by 1.4 and 1.5 after 12 weeks. However, as this study was lacking a low-altitude control group, it remains unclear whether the favorable effect was due to altitude exposure or the educational and exercise training per se.

In the present randomized trial, we could not show an additive effect of providing the rehabilitation at HA vs. LA on our primary outcome. This extends data from a previous non-randomized study suggesting that that allergen avoidance during HA sojourns improved bronchial hyperresponsiveness in adolescents with severe asthma [6]. Additional benefits of HA than mere allergen reduction have been postulated such as dryer, thinner and cleaner air, less fungi and an increased vitamin-D production due to increased exposure to sunlight [8].

In our study, the vast majority of participants (92%) had atopic asthma, but the improvements found with the rehabilitation at HA were similar as with the same program performed at LA. Nevertheless, we found a significantly greater reduction of FeNO in patients undergoing HA- vs. LA rehabilitation. This may point towards less airway inflammation in the HA-group. The findings are especially remarkable as FeNO measurements were performed at 760 m on the day after return from HA presumably when patients were already re-exposed to some allergens and other noxious stimuli. Our data are in line with previous reports of beneficial effects of HA rehabilitation in adults and children on FeNO and asthma control in both, allergic and non-allergic asthmatics according to nonrandomized studies [7, 11, 22].

Several randomized trials showed a positive impact of physical exercise and training on asthma control [5, 23–25] and maximal performance but no improvements in lung function [26]. Our study confirmed that a 3-week comprehensive rehabilitation program at both low and high altitude was highly effective to render sustained improvements in asthma control, quality of life and exercise capacity directly after the rehabilitation and up to 3 months.

We could show that exercise capacity assessed by the 6MWD and the STS improved significantly with our comprehensive 3-week rehabilitation program and the improvements were maintained up to 3 months follow-up, potentially due to the continuation of daily exercise according to the programs instruction in this highly motivated asthma collective. The improvement in exercise capacity found in both our groups where slightly smaller compared to other studies, assumedly because the rehabilitation lasted 3 weeks compared to 12 weeks [7]. Another study with a 12-week rehabilitation program at low altitude increased the exercise capacity in terms of the VO_2_ max and the total treadmill time [27]. Whereas the improvements in 6MWD were similar in the group that trained at HA vs. LA, we found a better improvement of the STS test in the HA vs. LA-group. This may point towards a higher effect of training at HA on exercise performance after return to LA. Such beneficial effects of altitude training have been previously described for many athletes and HA-training has been incorporated in training plans due to its increase in hemoglobin and thus oxygen transport [28]. Of interest, the additive effect of HA rehabilitation on the STS were maintained up to 3 months and might be at least partly explained by a significant increase in hemoglobin in the HA-group.

The significant improvement of asthma specific quality of life reflects an important effect of rehabilitation that was achieved to a similar degree in the HA and LA group. Also all sub scores of the AQLQ improved after the rehabilitation and remained at this level at 3 months follow up in both groups without difference between groups.

The program was well tolerated by patients at both locations, expect one patient at altitude who suffered from mild AMS (headache), no adverse events occurred. Intrinsically, it was not possible to blind investigators and patients to the intervention, and thus other factors than altitude and rehabilitation such as being away from home or factors concerning comfort might have played a role in the outcomes. However, this would be the case with any rehabilitation program all over the world. Among the participants of our study, there were no current smokers. Thus, our results may not be transferable to smoking asthmatics. The current study population revealed a relatively low and thus favorable daily PEF-variability and it may well be that the effect of a rehabilitation program including education and training would have been different, potentially even better, in a more compromised asthma collective. Despite these limitations, this study is of particular importance because of its robust design as a randomize trial.

## Conclusion

In the present randomized trial, we could show that a 3-week comprehensive asthma rehabilitation program including a clear action plan, peak flow diary, patient education, respiratory exercises, force and endurance training is highly effective in sustainably improving asthma control in terms of the Co-primary outcome asthma-related quality of life (ACQ) and PEF-variability, but also exercise performance and this positive effect was irrespective of the altitude is the rehabilitation program was performed.

## Additional file

Additional file 1: Asthma rehabilitation at high vs. low altitude: randomized controlled parallel-group trial. (DOCX 32 kb)

## Data Availability

The datasets used and/or analysed during the current study are available from the corresponding author on reasonable request.

## Abbreviations

6MWD: 6 min walking distance
ACQ: Asthma Control Questionnaire
AMS-c: Environmental Symptom Questionnaire cerebral score
AQLQ: Asthma-related quality of life questionnaire
asl: above sea level
FeNO: Exhaled nitric oxide
FEV1: Forced expiratory volume in 1 s
FU: Follow-up
GINA: Global Initiative for Asthma
HA: High altitude
IQR: Interquartile range
LA: Low altitude
PEF: Peak expiratory flow
SF-36: Short-form 36 questionnaire
STS: Sit-to-stand
