# Supplementary material for: Asthma rehabilitation at high vs. low altitude: randomized parallel-group trial
Source: BMC Pulm Med. 2019 Jul 24;19:134. doi: 10.1186/s12890-019-0890-y (PMC6657156; doi:10.1186/s12890-019-0890-y)
Supplement: Supplementary file 1 — Asthma rehabilitation at high vs. low altitude: randomized controlled parallel-group trial. (DOCX 32 kb) [file 12890_2019_890_MOESM1_ESM.docx]

# Asthma rehabilitation at high vs. low altitude: randomized controlled parallel-group trial

Stéphanie Saxer, Simon R. Schneider, Paula Appenzeller, Patrick R. Bader, Mona Lichtblau, Michael Furian, Ulan Sheraliev, Bermet Estebesova, Berik Emilov, Talant Sooronbaev, Konrad E. Bloch, Silvia Ulrich

**Online Data Supplement**

**Supplementary methods**

**1.1 Participants**

Patients were excluded if they had severe concomitant diseases precluding patients to adhere to the protocol and/or altitude exposure (severe metabolic, kidney- or liver disease, heart failure (ejection fraction < 50%), chronic lung diseases with a persistent FVC < 60% and /or peripheral oxygen saturation < 92%), severe mental- or musculoskeletal disorders.

**1.2 Intervention**

During the 3-week rehabilitation program, every patient had daily sessions for a total duration of 5hrs/day during 5 days a week in a rotating order in groups of 5 patients including the following topics:

- Patient education including:
  - Anatomy and physiology of the respiratory tract
  - Background medical information about asthma
  - Theory and practices about peak flow assessment and peak flow diary
  - Asthma action plan
  - Inhalation technique
  - Smoking cessation (if necessary)
  - Education about healthy life-style, daily activity and exercise
- Endurance training
  - Cycle ergometer with individual adapted resistance
  - Supervised with regularly measured heart rate and pulse oximetry
- Guided muscle strength training
  - Exercises of the upper and lower body (seated or standing)
  - Progression: exercises with elastic band
- Guided breathing exercises
  - Awareness of breathing pattern
  - Diverse breathing techniques in different positions were instructed
  - How to handle dyspnea
- Guided walks
  - Supervised walks outside (pulse and oxygen saturation control)
  - In groups of similar fitness

The rehabilitation program is comparable to that described by Rijssenbeek-Nowens and coworkers (1) and it reflects current guidelines suggesting that patient education physical activity (Evidence level A) and breathing exercises (Evidence level B) are important non-pharmacological treatments in asthma patients (2).

- 1. **Assessments**

The official Russian version of the asthma control questionnaire (ACQ) was used. The ACQ contains 7 items and the score ranges from 0 to 6, whereas a higher score reflects a worse asthma control (3). Question number 6 of the ACQ is about the use of reliever medication, which may be considered as exclusive indicator of asthma control according to guidelines and thus this item was analyzed also separately (2).

The AQLQ is a 32-items questionnaire including the following domains: activity limitation, symptoms, emotional function and environmental stimuli. A higher AQLQ means a better quality of life (4).

PEF was measured with a standard peak flow meter (vitalograph) four times per day and noted in patients diary. All patients participated in repeated patient education session to ensure correct PEF-meter usage and recording in the diary was daily supervised during the entire study.

Spirometry was performed with the device EasyOne (NDD, Zurich, Switzerland).

6MWT was done according to the ATS guidelines in a 30m corridor.(5)

The sit to stand test (STS) was done with a chair without armrests, patients had to stand up and sit down during one minute according to previous published studies.(6, 7)

FeNO was assessed by the NIOX vero (Circassia SA, Dietlikon, Switzerland) according to standard procedures.

Safety in the HA and LA-group was assessed by daily clinical examination and monitoring of PEF and symptoms. In the HA-group, patients additionally filled out the Environmental Symptom Questionnaire cerebral score (AMS-c) at the second day at altitude to assess symptoms of acute mountain sickness (8). The questionnaire was applied in Bishkek (760m asl) and in Tuja Ashu (3100m asl) and a cutoff point of ≥0.7 was defined as clinically relevant acute mountain sickness.

**References**

1. (GINA) GifA. Global Strategy for Asthma Management and Prevention, Global initiative for Asthma (GINA). . <http://wwwginasthmaorg/local/uploads/files/GINA_Report_2015_Aug11pdf>.

2. Global Initiative for Asthma Scientific C. Global Strategy for Asthma Management and Prevention 2017 [Available from: <http://ginasthma.org/2017-gina-report-global-strategy-for-asthma-management-and-prevention/>.

3. Juniper EF, Bousquet J, Abetz L, Bateman ED, Committee G. Identifying 'well-controlled' and 'not well-controlled' asthma using the Asthma Control Questionnaire. Respiratory medicine. 2006;100(4):616-21.

4. Juniper EF, Buist AS, Cox FM, Ferrie PJ, King DR. Validation of a standardized version of the Asthma Quality of Life Questionnaire. Chest. 1999;115(5):1265-70.

5. ATS statement: guidelines for the six-minute walk test. American journal of respiratory and critical care medicine. 2002;166(1):111-7.

6. Ozalevli S, Ozden A, Itil O, Akkoclu A. Comparison of the Sit-to-Stand Test with 6 min walk test in patients with chronic obstructive pulmonary disease. Respiratory medicine. 2007;101(2):286-93.

7. Puhan MA, Siebeling L, Zoller M, Muggensturm P, ter Riet G. Simple functional performance tests and mortality in COPD. The European respiratory journal. 2013;42(4):956-63.

8. Sampson JB, Cymerman A, Burse RL, Maher JT, Rock PB. Procedures for the measurement of acute mountain sickness. Aviat Space Environ Med. 1983;54(12 Pt 1):1063-73.

**Additional file 1: Table S1**

| **Use of reliever medication, Asthma Control Questionnaire question 6.** | | | | | |
| --- | --- | --- | --- | --- | --- |
|  | Baseline | End of rehabilitation  (3 weeks) | Follow-up  (3 months) | Between group difference BL-3w | Between group difference BL-3m |
| Low altitude | 1 (1; 2.5) | 1 (1;1)* | 1 (1;1) | 0 (-1 to 0) | 0 (0 to 1) |
| High altitude | 2 (1;2) | 1 (1;1)* | 1 (1;2) |  |  |
| *Note:* Data shown as median (quartiles).  *Abbreviations:* *: p<0.01 for Wilcoxon Baseline compared to 3 weeks | | | | | |
